# Role of Bedside Ultrasound in CMV Retinitis: A Case Report

**DOI:** 10.1155/2012/690598

**Published:** 2012-11-29

**Authors:** Lauren Westafer, L. Connor Nickels, Eike Flach, Giuliano De Portu, Latha Ganti Stead

**Affiliations:** ^1^College of Osteopathic Medicine, Nova Southeastern University, Fort Lauderdale, FL 33314, USA; ^2^Department of Emergency Medicine, University of Florida College of Medicine, Gainesville, FL 32610, USA

## Abstract

We present a case of retinal detachment diagnosed by emergency department bedside ultrasonography in a patient with CMV retinitis. The indications and findings of ocular ultrasonography are discussed.

## 1. Introduction

Retinal detachments, though uncommon, are devastating ocular emergencies that may result in permanent vision loss. While most retinal detachments are associated with age, myopia, inflammatory disorders, and trauma, individuals with human immunodeficiency virus (HIV) are at risk for CMV retinitis and subsequent detachment [1]. Individuals with CMV retinitis have an incidence of retinal detachment of approximately 50% per patient per year, as a result of the virus-mediated necrosis of the retina [2]. Although early detection of retinal detachment may preserve a patient's vision, CMV retinitis can infect both eyes and often progress to retinal detachment in days to weeks. As a result, practitioners should maintain a high index of suspicion for retinal detachment in HIV positive patients and use bedside ultrasound as a means of expedient evaluation in patients with visual changes.

## 2. Case

A 38-year-old male presented to the Emergency Department (ED) with a complaint of progressive vision loss in his left eye over the past week. The patient denied trauma and any prior ocular history. He denied photophobia, pain, discharge, pruritus, nausea, vomiting, or headache.

Physical exam revealed a pleasant, well-developed male who appeared comfortable. His past medical history was significant for HIV with an unknown CD4+ cell count. He was not on antiretroviral therapy but was undergoing treatment for toxoplasmosis. On exam, he had no facial swelling, erythema, or discharge from his eyes. His conjunctivas were injected bilaterally. His extraocular movements were intact. The patient's pupils were round bilaterally, but his left pupil was nonreactive to light. There was no pain with movement of his extra-ocular muscles. He reported complete loss of vision in his left eye, including inability to perceive light. Visual acuity in the patient's right eye was 20/200, his reported baseline.

**Figure 1:**
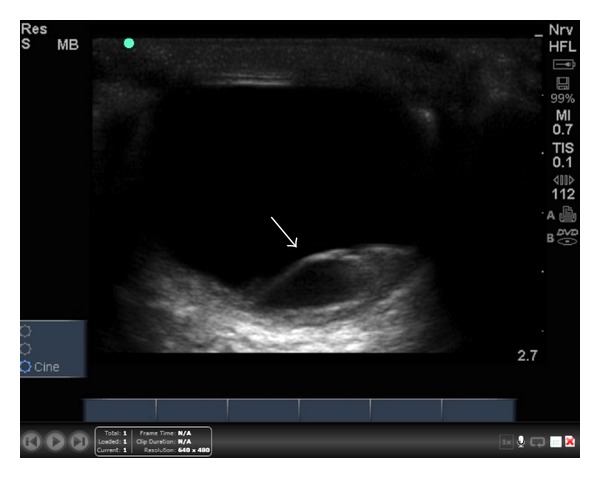
Left eye ultrasound shows retinal detachment.

**Figure 2:**
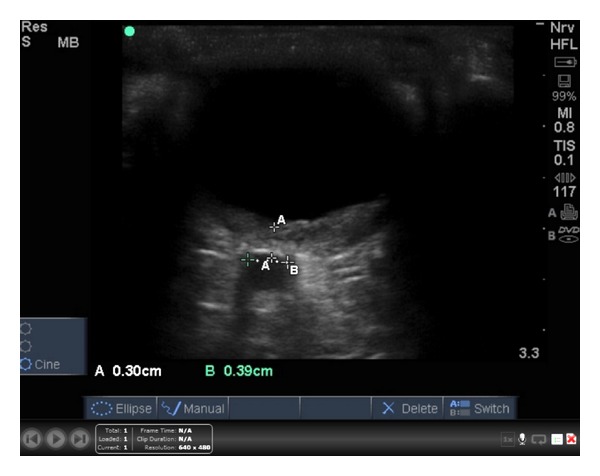
Left eye optic nerve sheath measurement, consistent with normal ICP. (Below).

 A high frequency 7.5–10-MHz linear array transducer was used to perform the ocular examination. A large amount of standard, water-soluble ultrasound gel was applied to the patient's closed eyelid. The patient was instructed to look straight ahead. The eye was scanned in both the sagittal and transverse planes, using essentially no pressure on the globe. The ultrasound demonstrated a large retinal detachment in the left eye with no macular sparing (Figure 1). No vitreous hemorrhage was detected, and findings of elevated intracranial pressure were not present (Figure 2). Ultrasound of the right eye demonstrated no retinal detachment and a normal globe. Ophthalmology was consulted and asked to see the patient in the outpatient clinic. The patient was diagnosed with CMV retinitis in his bilateral eyes. He was treated with intravitreal Foscarnet, Pred-Forte (prednisolone acetate) eye drops, and Vigamox eye drops. At two-week followup, vision in his right eye was improving, and no retinal detachment had developed in that eye.

## 3. Discussion

Retinal detachment lacks obvious physical findings and may result in monocular blindness, especially if missed. Historically, the gold standard diagnostic examination for retinal detachment is a thorough ophthalmoscopic evaluation, but this is often not immediately feasible due to consultant availability, dilation contraindications and equipment, or medication limitations [3, 4].

Ophthalmologists have used ultrasound for decades. Retinal detachment can be described as a thick, hyperechoic membrane that appears dissociated from the posterior segment of the eye. This membrane is often described as undulating and may be difficult to detect if the retinal detachment is small [4].

Emergency physicians have been using ultrasound to correctly diagnose retinal detachment for over a decade [5]. Recently, more studies have validated the limited training requirements needed for emergency physicians to accurately detect retinal detachment [6, 7]. Studies vary, with reports of twenty minutes to one-hour lectures followed by hands-on practicums. As a result, bedside ultrasound is gaining momentum in the diagnosis of this condition. Prospective studies in the Emergency Department have demonstrated that emergency physicians have 97–100% sensitivity in diagnosing retinal detachment using ultrasound. The specificity is not compromised, with studies reporting specificities of 92% [4, 8, 9]. The literature also has shown that bedside ultrasonography has been able to correlate nerve sheath diameter (ONSD) with abnormalities in intracranial pressure [10].

The most emergent retinal detachments are when the area of the macula is not detached, termed a “mac-on” retinal detachment [5]. Detached retinas that preserve the area of the macula can benefit from early intervention and allow the patient to regain their predetachment vision. Unfortunately, delays in diagnosis and intervention result in a progressive condition that eventually involves the macula, leaving the patient with a permanent deficit [11]. Use of bedside ultrasound allows emergency physicians to quickly diagnose and risk-stratify patients with a potential ocular emergency. In the case of our patient, the vision in his right eye was preserved as a result of detection and treatment. Evaluation of the ONSD showed a 3.9 mm measurement consistent with normal ICP [10]. Furthermore, emergency physicians detecting a “mac-on” retinal detachment may be able to advocate for quicker evaluation by ophthalmology or transfer to a facility capable of ophthalmologic intervention. Further investigation is needed to determine the ability of emergency physicians to differentiate between “mac-on” and “mac-off” detachments.
